# Vaccine injury compensation: the Israeli case

**DOI:** 10.1186/s13584-021-00490-w

**Published:** 2021-09-13

**Authors:** Shelly Kamin-Friedman, Nadav Davidovitch

**Affiliations:** 1 Department of Health Policy and Management, School of Public Health, Faculty of Health Sciences, Ben-Gurion University of the Negev, Be’er Sheva, Israel; 2 Faculty of Law, Haifa University, Haifa, Israel

**Keywords:** Vaccination, Vaccine, Social welfare-oriented legislation, Compensation, Fairness, Solidarity, Trust

## Abstract

**Background:**

Following other countries, Israel passed the Vaccine Injury Compensation Law in 1989, which provides for compensation to vaccine recipients who had suffered injuries without proving negligence. In 2021, after deliberations between the ministries of health and of finance Covid-19 vaccines (administered from the beginning of the campaign on December 20, 2020 and up to December 21, 2022) were included within the compensation law. The current study aims to examine the objectives of Israel’s Vaccine Injury Compensation Law, at the time of its enactment, and to explore barriers to their fulfillment. These issues are especially relevant in light of the discussions held on the option for liability exemption which excludes the possibility of redress from the Covid-19 vaccine manufacturers in case of injury attributed to the vaccine, and considering the heavy burden of proof required in standard tort law.

**Methods:**

The study employed a qualitative methodology which made use of both content analysis of relevant documents and in-depth interviews.

**Results:**

In passing the Vaccine Injury Compensation Law, legislators sought to assist vaccine recipients who had suffered injuries by both lowering their burden of proof as well as establishing a short and efficient procedure for deliberating their claims. Furthermore, legislators believed that the assurance of compensation to vaccine recipients who had suffered injuries would help to encourage a high rate of vaccination compliance. An examination of the law’s implementation over time revealed that the aforementioned goals were not attained.

**Conclusions:**

Implementation of the law since its enactment missed the opportunity to fulfill its original purposes to promote public health fundamental principles of fairness and solidarity. In addition, the adversarial proceedings as well as some of the law’s provisions have the potential to undermine public trust in the State’s willingness to grant compensation for injuries that are attributed to vaccines and thereby subvert the law’s pivotal objective of promoting trust and vaccine compliance. We suggest that allowing circumstantial evidence as to an association between vaccine and an injury, transitioning to administrative deliberation, making available to the public details of cases where compensation was awarded, as well as other possible emendations would help it better reflect the values of fairness and solidarity that underlying the law's purpose. These would also promote the level of trust in healthcare authorities which is essential to preserving high vaccine coverage.

## Introduction

The COVID-19 pandemic, which began spreading globally in early 2020, had infected over 216 million people as of August2021.

Its high burden around the globe led to the expedited development of vaccines by a number of mostly private manufacturers, along with purchase agreements between manufacturers and governments [1]. As reported in the media [2], the agreements Israel has signed with American manufacturers of COVID-19 vaccines include a “liability exemption” clause for the manufacturers in the event of adverse effects.

The alleged liability exemption given to Covid-19 vaccine manufactures by the State of Israel requires an examination of the legal remedies for injuries that are attributed to vaccines according to Israeli law.

On January 5, 1988, then-Knesset member Haim Ramon submitted a bill for compensating vaccine recipients who had suffered injuries. Following deliberations by the Knesset Labor and Welfare Committee, the Vaccine Injury Compensation Law was passed in 1989 [3, 4].

The background of Israel’s legislation regarding compensation for vaccine injuries from the court’s recommendations to the legislator to find a legal way to ensure compensation for vaccine recipients who had suffered injuries, as these claims are mostly dismissed according to torts law. In 1986, Juhar Altouri (represented by her guardian) filed a claim against the Ministry of Health, claiming that after receiving a trivalent vaccine against diphtheria, tetanus and pertussis, she suffered high fever, loss of appetite, right facial palsy, loss of reflexes in the right leg and weakness in the left hand. She was later diagnosed with severe encephalitis. The illness left her with mental retardation and mild paralysis of the right torso. Juhar Altouri based her claim on the State’s alleged negligence in administering the vaccine. It was argued that the infant had been administered a defective vaccine, which was known to have caused damage in the past, but, nonetheless, had not been tested. The claim also asserted negligence on the part of the State for failing to warn the child’s parents of risks associated with the vaccination. The District Court dismissed the claim, ruling that there had been no negligence in administering the vaccine, and that no causal link could be established between the vaccine and the child’s injuries. The claim regarding failure to provide adequate information on vaccine risks was also dismissed, owing to lack of evidence.

On appeal, in 1993, the Supreme Court of Israel upheld the decision to dismiss the claim. The court ruled that administering the vaccine could not be deemed negligent because the benefits to public health outweighed any risks possibly associated with the vaccine. Likewise, a causal connection could not be proven between the vaccine and the injuries, based on the timeframe of the vaccine’s administration and the onset of symptoms alone. However, the ruling also suggested, that in the absence of a tort solution, the legislature should consider formulating a law that will allow vaccine recipients who had suffered injuries to obtain compensation from public funds [5]. Although this Supreme Court ruling was issued after Israel’s Vaccine Injury Compensation Law, 1989, had already been enacted, the full course of the litigation took place concurrent to the legislative process and is therefore relevant to understanding the legislative background in Israel.

A similar statement to that of the Supreme Court in the Altouri case had been issued a few years earlier at the Haifa District Court. The court recommended that the legislature consider enacting a law that would grant compensation to the small number of vaccine recipients who had suffered injuries and faced difficulties in proving a clear causal link between vaccines and injuries incurred, considering the heavy burden of proof required in standard tort law. “Such a law, which would have social objectives, could adopt a much more liberal standard than that in ordinary tort law” [6].

According to the law, the State would insure all people receiving vaccines, and anyone with whom they came in contact, for injury resulting from vaccination. The law specifies the insured vaccines: DTP, polio, MMR, Hemophilus influenza B, hepatitis B, and any other vaccine administered in accordance with section 19 of the Public Health Ordinance, 1940 (which relates to obligatory vaccines in cases of hazardous epidemics). Injuries resulting from these vaccines would be entitled to compensation, provided the vaccines were not given as part of clinical treatment.

On February 4, 2021, after deliberations between the ministries of health and of finance, the Israeli legislator added Covid-19 vaccines (administered from the beginning of the campaign on December 20, 2020 and up to December 21, 2022) to the list of insured vaccines.

The Vaccine Injury Compensation law holds that it is not necessary to prove negligence on the part of the vaccine provider or other party in order to receive insurance benefits in the event of injury. In the case of a vaccine injury claim being submitted according to the law, a committee of experts, including a judge and two physicians, are to determine whether there was a causal connection between the vaccine and the injury, as well as the extent of permanent disability. The committee’s decisions can be appealed in district courts.

An individual bringing a claim under this law is not entitled to submit an additional claim under the Torts Ordinance. A tort claim can only be submitted as an alternative to a claim submitted under the law. On October 25, 2020, the Israeli Supreme Court held that in tort claims in which claimants failed to prove negligence, the State cannot then be forced to compensate them by virtue of a “constitutional cause”, that is, due to an argued violation of the constitutional right for legal remedies, as there is an alternative no-fault compensation arrangement stipulated by the Vaccine Injury Compensation Law. Thus, compensation without proof of negligence can be paid only in the context of the Vaccine Injury Compensation Law [7].

Vaccine Injury Compensation Regulations issued in 1992 [8] established an insurance fund for vaccine recipients who had suffered injuries, which names the Internal Government Insurance Fund, managed by Inbal Insurance Company Ltd., as the “insurer.” The “insured” are the Ministry of Health, health funds (*kupot holim*), municipalities, or physicians administering vaccinations; the “beneficiaries” are the person receiving the vaccine from the “insured” and anyone with whom that person comes in contact. Since legal claims are, in fact, insurance claims, the statute of limitations is three years (for minors, three years after reaching age 18).

The former legally appointed Chairman of the Committee, honorable judge Dr. Bilha Kahana, who has served for most of the period the Committee has operated, observed in 2008 that the Committee had never adjudicated compensation for claimants who claimed vaccine injury according to the Vaccine Injury Compensation law. In those cases in which compensation was paid, it was given *ex gratia* as part of a settlement [9]. Similarly, examining Israeli latter judgements reveals that the expert committee appointed according to the law did not adjudicate compensation between 2008 and 2021.

## Study objectives

The current study aims to examine the objectives of Israel’s Vaccine Injury Compensation Law, 1989 at the time of its enactment, and to explore barriers to their fulfillment. These issues are especially relevant in light of the discussions held on the option for liability exemption which excludes the possibility of redress from the Covid-19 vaccine manufacturers in case of injury, and considering the heavy burden of proof required in standard tort law.

## Methods

The study themes relating to Israel’s Vaccine Injury Compensation Law objectives and implementation are multi-faceted and require thorough discussion. Therefore, it was decided to conduct a constructive, qualitative study, which is not limited to closed questions [10, 11].

### Data collection

Data collection began by reviewing relevant documents, such as protocols from Knesset debates, court judgements and legislation. In the following phase, in-depth individual interviews were conducted, based on predetermined general guidelines (“Appendix”). The interviews were conducted with 13 informants from a number of disciplines: two attorneys involved with vaccine injury claims, one who represents Inbal, the governmental insurance agency, and one who represents claimants who claim vaccine injuries; an attorney who had reviewed the Vaccine Injury Compensation Law on behalf of the Israeli Bar Association; a retired judge who presided over cases of the appointed committee; a physician specializing in public health and employed by the Ministry of Health; two public health researchers; a physician who treats patients who have suffered adverse vaccine effects; three claimants who allege having suffered vaccine-related injuries (via legal guardians); and two jurists specializing in healthcare ethics.

The sample of informants was deliberately selected to allow for maximum variability in attitudes to the research themes. The assumption underlying the sample selection was that medicine, law and ethics were the relevant fields for evaluating a law on vaccine-related injuries. The opinions of vaccine recipients who had suffered injuries were also relevant, and their views are crucial to any discussion on vaccine injury compensation.

## Research tools

The qualitative research tools included content analyses of relevant documents [12], which were followed by in-depth interviews with the research informants [11]. The interview questions were based on predetermined general guidelines (see “Appendix”).

The issues raised in the general guidelines were selected with the aim of uncovering the manifold aspects of the research from the informants’ diverse perspectives, and were based on the critical analysis of vaccine injury compensation laws in other countries [13, 14].

The order in which the issues were presented varied between interviews, so as not to interrupt informants who wished to speak more extensively on certain topics or who introduced new issues of their own accord. Informants were also encouraged to expand on issues they raised on their own initiative, without disrupting the flow of the conversation. Significant topics introduced by informants were incorporated into the general guidelines for later interviews.

### Data analysis

Documents and protocols documenting the in-depth interviews were read in full during the first phase of analysis, and the main issues were highlighted. During the second phase, a Word document was prepared, and the units of meaning from each protocol and document were copied into it. During the third phase, the units of meaning were organized according to inclusive thematic categories. The fourth phase consisted of a comprehensive rereading of the protocols and documents, to ensure that data did not diverge from their original meaning in the construction of categories [11]. After analyzing the thirteenth interview, no new thematic categories emerged, thus leading to our conclusion that we had reached data saturation.

Research conclusions were based on inductive analysis of the data gathered from the documents and interview protocols, according to grounded theory strategy [11].

## Results

The presentation of the study results consists two parts. The first part relates to the law objectives, as emerged from analyzing the content of legislative documents, including the Vaccine Injury Compensation Bill, and protocols documenting the legislative process in the Israeli Knesset.

The second part examines the question of whether the objectives of the law have been achieved and explores barriers to their fulfillment, according to the positions of the informants as emerged from the in-depth interviews.

### Objectives of the Vaccine Injury Compensation Law, 1989

The explanatory notes to the Vaccine Injury Compensation Bill, 1988, open with the following sentence: “Aid to those injured as a result of mass vaccination policies has not yet been regulated by law” [15]. This statement indicates that the legislature’s primary objective was to aid vaccine recipients who had suffered injuries. As will be clarified below, the legislature sought to aid the injured both in attaining monetary compensation and in streamlining and expediting the legal procedures involved.

#### Compensating vaccine recipients who had suffered injuries

During the 11th Knesset’s deliberations on the bill (on January 5, 1988), the goal of “allowing compensation for vaccine recipients who had suffered injuries" was explicitly set forth [16], and during the deliberations of the Knesset’s Labor and Welfare Committee (May 24, 1988), Knesset member Haim Ramon declared: “I have proposed a bill which will instate a mechanism to compensate the injured individuals” [17].

The justifications for awarding compensation to vaccine recipients who had suffered injuries were as follows:

- Vaccination was in accordance with government policy: “Today, the situation is that children or adults who suffer disability or physical, mental or emotional impairment as a result of a vaccine administered as part of state policy are forced to…submit their case to court” [15].
- Vaccination was an action for the common interest: “Vaccine coverage is a public interest of primary importance, it is done for the greater good and therefore there should be public liability for injury vaccines may cause to individuals” [16].
- Vaccination may cause severe injuries, and there is a small number of cases in which such injuries occur: “Vaccine-related injury, especially that due to the trivalent vaccine, is generally severe – death, severe mental retardation, 100% disability, deafness, muteness, and so on. Often the parents also suffer… caring for a child with disabilities may lead to severe financial crisis within the family, on top of the personal crisis”; “Every year, a small number of children incur injuries from vaccines…I think there is little concern that there will be a flood of lawsuits due to the small number of injured [vaccine recipients] each year…compensation should be provided for that small percentage of people” [16].

To the Israeli legislators, aiding vaccine recipients who had suffered injuries to obtain compensation justified a law that would ease the burden of proof. It was clarified that claims filed as negligence-based tort actions would be dismissed, due to the difficulty of proving both negligence and a causal connection between the vaccine and the injuries incurred: “In every population of infants, a certain number of infants are sensitive to the vaccine. Their sensitivity cannot be determined until they receive the vaccine. No one is guilty” [16];”As things stand today, a person injured as a result of vaccination, child or adult, must file a tort claim for compensation in civil court. To win his claim, the claimant must prove two things: one—a causal connection between the vaccine and the injury, and two—negligence in vaccine administration or a defective vaccine. In the vast majority of cases, it is nearly impossible to prove the two aforesaid factors in court proceedings, and the outcome is that the person injured, who often suffers severe injuries, up to 100% disability, is left without any compensation” [18].

#### Short and effective deliberation on vaccine injury claims

In addition to guaranteeing compensation for vaccine injury, the legislature also sought to aid the injured individuals by ensuring that their claims would be adjudicated expeditiously. During Knesset plenary deliberations on the bill, the injustice suffered by vaccine recipients who had suffered injuries as a result of prolonged and costly court proceedings was emphasized: “Such claims remain pending in court for years. They involve a lengthy and expensive judicial process…An enormous burden on all parties [is] involved to conduct a complex, costly and lengthy trial, requiring lengthy and complicated inquiry of experts. The courts view the imposition of such a burden on private citizens as unjust, considering the circumstances surrounding the children’s injuries” [16].

In accordance with the bill’s explanatory notes, the Committee of Experts established under the law is meant to facilitate “efficient and expeditious hearings on vaccine injury claims” and protect the families from “long and costly legal proceedings.” In its final clause, the explanatory notes highlight the necessity of “immediate compensation” for vaccine injuries [15].

#### Promoting vaccine compliance

Israel’s Vaccine Injury Compensation Law was motivated not only by the aforementioned objective to aid vaccine recipients who had suffered injuries but also by the objective to promote vaccine compliance. Assured compensation and expeditious judicial proceedings would serve to encourage vaccination compliance, rather than being end goals in and of themselves.

It is in this context that the explanatory notes to the Vaccine Victims’ Insurance Bill state that, “The importance of maintaining immunization coverage in Israel, which is among the best in the world, requires enacting State responsibility and immediate compensation to individuals affected by this practice” [15].

### Were the Law’s objectives achieved?

As aforementioned, at this second part of the research the law objectives were presented to the informants, and the questions of whether the objectives have been achieved or what are the barriers to their fulfillment were examined by in-depth interviews.

As to the law objective of compensating vaccine recipients who had suffered injuries, the fact that the Committee of Experts has never ruled in favor of compensating vaccine recipients under the Vaccine Injury Compensation Law [9] led the majority of the study’s informants to conclude that the law had failed: “I think that the law, in the end—and actually many agree—did not live up to expectations…practically speaking, it is a resounding failure because it is not employed often enough” (public health researcher).

This study’s informants clarified that the evaluation of a causal connection between the vaccine and injuries according to scientific criteria is what led to the dismissal of claims: “One of the things that makes it difficult is the whole issue of the complexity of causation claims…even if it…may be linked to the vaccine, it could also be linked to a thousand other things that happened at the same time, and therefore it’s very hard to say what can be attributed directly to the vaccine…all the research around the world shows that all the side effects in question are not related to the vaccine but to other things…I need to act according to the science, not feelings” (a physician specializing in public health, employed by the Ministry of Health).

Another respondent, a retired judge who presided over cases of the appointed committee, echoed these observations: “In the claims submitted to me, no one was compensated because no one managed to pass the bar of proving a causal link…and this, in my opinion, is one of the law’s faults.”

In addition to the above specificity requirement of the connection between the vaccine and the injury mentioned by the informants, scientific causality is examined according to additional criteria: strength of association, consistency, temporality, biological gradient, plausibility, coherence, experiment, and analogy (the “Bradford Hill" criteria) [19]. When it comes to vaccine damages, the difficulty in proving the above criteria for scientific causality leads to the dismissal of claims filed under the law, similar to the dismissal of claims arising under tort law that also require scientific causation.

Some of the informants suggested that “easier criteria” should be required to make a legal showing of a causal link than those required for drawing scientific conclusions. The reasoning behind this view was that a causal link needed to be established for legal rather than medical purposes: “The scales should lean in the direction that there is a reasonable likelihood of a causal link…after all, we know that there is a difference between causal links in law versus in epidemiology or public health” (an academic public health specialist). Another justification for reducing the difficulty of proving a causal link was that the law’s goal was to assist vaccine recipients who had suffered injuries in receiving compensation: “You have a country that wants to help those who suffered harm as a result of going and doing something for the good of the country, and all it wants to know is whether there is a high likelihood [of causation] and nothing more; it’s more likely than unlikely that [the injury] was caused by the vaccine, and in my view, this should be thoroughly sufficient” (attorney who represents claimants who claim vaccine injuries).

Among the ways for facilitating the establishment of a causal link, it was suggested that its determination could rely on circumstantial evidence as to an association: “I say we should get away from epidemiological causal links, and rather use common sense…" (public health researcher).

For cases in which a causal link between the vaccine and injury is proven, the Vaccine Injury Compensation regulations stipulate fixed compensation rates based on the degree of permanent disability. No compensation is awarded in cases of temporary disability, nor is there restitution for lost workdays, medical treatment, or third-party assistance.

Some informants voiced the opinion that the maximum sum set by the regulations does not correspond to the harm caused by severe vaccine-related injury: “In the end, if someone is really harmed, and we’re usually talking about significant harm from a vaccine, which may be neurological damage or even death…then because the State was afraid…and wanted to establish some sort of limit, this aspect is really inadequate” (public health researcher).

It was also argued that social legislation should compensate victims for temporary injuries: “In social legislation, compensation is provided for emotional suffering too. Say, if a person suffered side effects and was hospitalized, and that caused him suffering…he experienced trauma even if he fully recovered. And here, there’s no compensation for that…so there is no restitution for the hospitalization and suffering, lost workdays, anxiety and so on. I think that this is another problem with the law” (an academic public health specialist).

Finally, several informants noted that the statute of limitation under the law, of three years from the date of vaccination (or three years from the moment an injured minor reaches adulthood), also hinders the law’s objective to compensate recipients who had suffered injuries. They claimed that it was morally inappropriate to even set a statute of limitation considering the law’s social objectives: “There are administrative decisions. But morally speaking, if new information comes to light and you want to know the truth, should it matter how much time has passed?” (a physician who treats patients who have suffered adverse vaccine effects); “If you have a law, and it’s a social law, I think you should accommodate the injured individuals as much as possible… And that goes for the statute of limitation too” (a jurist specializing in healthcare ethics).

On the other hand, it was claimed that it is imperative to limit the period in which claims can be filed, in order to ensure the insurance company stability and financial security, and in light of the challenges facing the insurance company in uncovering the relevant evidence long after the vaccination was given.

Considering the Vaccine Injury Compensation Law’s objective to enable expeditious and efficient deliberations on claims, informants noted the challenges to filing a claim due to the lack of a clear and simple procedure and the informal demand to include an expert opinion with the claim. As one informant argued: “Not everyone has legal expertise or understanding, not everyone is an academic and not everyone knows how to proceed. They are in a difficult emotional state. For all these reasons, they should be able to fill out the simplest form in the world: I claim that on this and this day my son was injured after receiving a vaccine, then add a medical document that shows the clinical record” (an attorney who represents claimants who claim vaccine injuries).

In addition to the challenges in filing a claim, informants noted that deliberating claims in an adversarial procedure placed an additional obstacle to obtaining an expeditious and efficient hearing.

The law stipulates that the Committee of Experts will discuss claims and determine whether there is a causal connection between the vaccine and the injury, as well as the degree of disability. The law fails to address whether the committee is subject to the rules of evidence and procedures customary in court proceedings. However, informants clarified that, in practice, claims filed under the statute followed procedures similar to those in adversarial civil tort claims. They noted that a possible reason for employing the adversarial format was the presence of lawyers representing the Inbal Insurance Company, which manages the Internal Government Insurance Fund. The law does not specify any role for the Inbal company other than the payment of compensation to the injured parties in accordance with the Committee of Experts’ decision. Despite this, in practice, Inbal lawyers represent the State in presenting the defense arguments to the Committee of Experts: “Maybe here it would be preferable to not have…[legal] representation of Inbal. That is, I don’t know exactly what their influence is in this game…They serve as the State’s insurance company in this issue…It’s problematic…because it turns it back into a legal process” (public health researcher).

Relating to the law's objective to promote vaccine compliance, the informants noted that the adversarial proceeding, which is conducted as a confrontation between the injured individual and the Ministry of Health’s insurance company also undermines the public’s trust in the Ministry of Health (in addition to its negative effect on expeditious proceedings).

Trust diminishes since the deliberation does not allow for consideration of aspects of the injury other than the pecuniary damages: “In an adversarial procedure, the parties facing the family generally won’t give space to issues of emotional suffering and trauma” (public health researcher).

Moreover, the adversarial procedure inevitably places the State in the position of the injured “opponent,” giving them the feeling that the law is a mere token gesture: “I don’t really believe there can be trust-building in an adversarial procedure…I think that there are more—let’s call them conciliatory procedures, which I think would contribute to all parties involved and build trust” (public health researcher); “As long as the procedures are adversarial, I think the public feels that the law is just lip service, rather than something the State really stands behind” (a jurist specializing in healthcare ethics).

A legal guardian of a minor who claims to have suffered a vaccine-related injury added: “Why do I have to confront someone who doesn’t want to pay me?…It means that either I have to hire a lawyer at my own expense, which costs more than what I might receive here, or I have to show up alone and face some shark lawyer who’ll eat me alive, and that’s fine with everyone. What are they actually doing there?”.

Public confidence in the health authorities’ willingness to compensate vaccine recipients who had suffered injuries has been undermined also because of the law’s provision regarding choice of jurisdiction for bringing a claim, either through the Vaccine Injury Compensation law, or through a tort claim in court. A legal guardian of a minor who alleges having suffered vaccine-related injury noted: “They seem to be silencing [people] in a way…They hand you the compensation, but then you don’t have the option to sue the State even though it is responsible.”

The law’s lack of a requirement to publish cases in which compensation was given (by settlement as well), was also noted as a cause for undermined public trust in the health authorities. Committee deliberations on vaccine injury compensation are held in the Jerusalem Magistrate’s Court, and its judgements are published in Israel’s legal databases under the heading of “various civilian claims”. However, claims that result in monetary settlements *ex gratia* are not published at all, and people receiving compensation must confirm in writing that any compensation given to them does not constitute acknowledgement on the part of the State of a causal link between the vaccine and injury: “Some of the mistrust arises from concerns that information is being concealed…If the Ministry of Health’s decisions in vaccine injury claims were fully disclosed…I think this would reduce the sense of alienation between…vaccine policy and the public. I think that in order to create a less alienating atmosphere, improve trust between the public and the Ministry of Health, and avoid suspicions of conspiracy and so on, which sometimes arise among the public, I think it’s preferable to publish them” [settlements for compensating vaccine recipients who had suffered injuries] (a physician specializing in public health and employed by the Ministry of Health). On the other hand, concerns were voiced that knowledge of this compensation might deter the public from getting vaccinated.

## Discussion and conclusions

Israel’s Vaccine Injury Compensation Law was enacted to advance the moral objectives of fairness and solidarity and to promote the practical goal of encouraging vaccine compliance. The principle of fairness demands that individuals be compensated for injuries incurred as a result of choosing to be vaccinated to benefit the greater good. Moreover, in accordance with the principle of fairness, if the State decides on vaccination policy, it should also be obligated to compensate individuals injured as a result of such policy [20, 21].

Principles of solidarity demand that each individual must bear equally the risks borne by the community, including risks that arise from vaccines against infectious diseases. Thus, the burden of vaccine-related injury, which is not evenly distributed among vaccine recipients and *a fortiori* among the entire population, should entitle vaccine recipients who had suffered injuries to compensation. The principle of solidarity also obligates the community to provide support for individuals, so that no one person should individually bear the burden of injury [20, 22, 23].With regard to the utilitarian considerations of promoting vaccine compliance, it has not been empirically proven that compensating injured individuals through legislation improves vaccine compliance [20]. However, legislation to compensate vaccine recipients who had suffered injuries attributed to the vaccine (assuming it does in fact aid the injured individuals) has the potential to bolster trust between vaccine recipients and the healthcare system [24]. There are also studies demonstrating that trust in health authorities predicts vaccine compliance [25].

The legislature’s waiver of the requirement of proving negligence to obtain compensation for injuries that are attributed to vaccines eased the burden of proof on the claimants. However, the strict requirement to prove a scientific causal link between vaccine and injury presents a serious obstacle to the claimants and thus does not advance the principles that underlie the law, i.e. fairness, solidarity and trust. It therefore may be beneficial to consider the suggestion raised by informants in the course of this study to ease the criteria for determining a causal connection between vaccination and subsequent injuries. Easier criteria might be presumptions for causal connection between vaccines and known injuries, as in the Vaccine Injury Compensation Program in U.S. law [26], and/or allowing circumstantial evidence for the establishment of causal connection.

A discussion about the possibility of proving a causal connection based on circumstantial evidence rather than scientific evidence was held by the Committee of Inquiry in 2001 into the health consequences of Kishon River military operations among Israeli Defense Force soldiers. The committee, composed of a judge and two medical experts, was tasked with determining whether exposure to the contaminated Kishon River water had caused a surge in cancer cases among soldiers who had trained in the area. In statistical tests, no significant difference in morbidity was found between soldiers exposed to Kishon River water and those who had not been exposed.

However, Committee chairman and retired Supreme Court President, the late Meir Shamgar, stated (in the minority opinion) that “statistical conditions do not necessarily need to overlap with reasonable causal connection… Cumulative reason, logic, analogy and experience also indicate a causal connection.” Judge Shamgar noted that in tort claims, a causal link is established if the probability is greater than 50% and thought that the committee must examine the various theories presented and accord one of them preferential reasonableness. Judge Shamgar considered a statistical epidemiological test of scientific significance, but limited in terms of providing insights into the data as a whole. Contrary to Judge Shamgar’s views, the medical experts who reviewed the issue of injuries to divers after Kishon River exposure concluded that it had not been scientifically proven that the river’s contamination caused a significant increase in cancer incidence. However, since cancer-causing substances are known to pollute the Kishon River, and since there was an increased incidence of cancer among the exposed divers, it was recommended *ex gratia* to recognize the soldiers as having fallen ill during and due to their military service [27].

In 2004, the Israeli Supreme Court in CA 1639/01 Kibbutz Maayan Zvi v. Yitzhak Krishov [28] reinforced Judge Shamgar’s minority opinion and decided on the existence of a causal link based on circumstantial evidence. The court accepted the argument that Non-Hodgkin’s Lymphoma had been caused due to exposure to asbestos at the workplace, despite the fact that no medical studies have unequivocally proven a causal connection between exposure and the illness.

Later Israeli judgement CA 6102/13 Michael Azmon v. Haifa chemicals [29] clarified that scientific evidence is required to establish causation, excluding the unique cases where medical research has not yet found or ruled out casual connection. In these circumstances only, causality may be established according to circumstantial evidence.

As the Vaccine Injury Compensation Law has a social objective to assist vaccine recipients who had suffered injuries, it is more than reasonable to suggest that the experts committee appointed according to the law would allow circumstantial evidence in order to establish causality when there is no clear medical evidence for or against causation.

This approach resembles the opinion of the Court of Justice of the European Union (ECJ) regarding liability of the producer of a vaccine due to an alleged defect in that vaccine: “In the exercise of its exclusive jurisdiction to appraise the facts, (the court) may consider that, notwithstanding the finding that medical research neither establishes nor rules out the existence of a link between the administering of the vaccine and the occurrence of the victim’s disease, certain factual evidence relied on by the applicant constitutes serious, specific and consistent evidence enabling it to conclude that there is a defect in the vaccine and that there is a causal link between that defect and that disease” [30].

Another way to promote the law’s objectives would be to compensate vaccine recipients who had suffered injuries attributed to the vaccine not only for their pain and suffering due to permanent disability, but also for their loss of income, third-party assistance or medical treatment costs. It is worth noting that the Road Accident Injury Compensation Law, for example, which, like the Vaccine Injury Compensation Law, grants compensation without proof of negligence, provides compensation for all actual damages and is not based merely on the injured degree of disability. The legislature must consider whether there should be a distinction between a law of compensation for road accidents injuries and a law of compensation for vaccine injuries, when both laws were legislated with social objectives—to assure compensation for every injured. Nonetheless, it should be noted that the Road Accident Injury Compensation Law sets strict ceilings for compensation for non-pecuniary damage and for loss of earning and earning capacity. It is therefore doubtful whether it has fulfilled its social objectives [31].

Expeditious litigation as a means of assisting claimants and thus bolstering fairness, solidarity and trust in health authorities may be achieved by deliberating claims in an administrative procedure, as opposed to adversarial proceedings.

The adversarial method by which lawsuits are currently reviewed, requires claimants to prove their claims through a confrontation, with the State essentially a counterparty. As a result, the injured parties feel the need to employ legal counsel and pay for medical opinion, which make the procedure more expensive. Cross-examination carried out by both parties prolong the legal procedure, which can then last as long as do tort litigations in court. The adversarial proceedings have also been criticized in the literature, for evoking antagonism toward the state on the part of the claimants [13].

An administrative procedure may allow members of the Committee of Experts to arrive at their conclusions based on clinical records and bolster expeditious judgements. Additionally, under an administrative procedure, members of the Committee may be granted the authority to actively investigate claims, and thus it would be unnecessary for the state to have legal representation. Waiving the presence of a legal representative has the potential of diminishing confrontation between the injured and the health authorities and thus the potential of promoting trust.

The majority of no-fault vaccine injury compensation programs legislated in the World Health Organization Member States stipulate that the discussion of vaccine injuries will be conducted in an administrative procedure. Proponents of administrative procedure claim that it allows fairer access to compensation for vaccine recipients who suffered injuries [22]. In the USA for example, with a combination of an administrative and legal approach [32], the percentages of vaccine-related claims awarded monetary compensation (for petitions filed through 10/01/1988 through 09/01/2020) were as follows: 36% due to the hepatitis B vaccine; 32% due to the DTP vaccine; 38% due to the Hemophilus Influenza B vaccine; 38% due to the MMR vaccine; and 46% due to the hepatitis A vaccine [33].

It is important to note that deciding that causal connection can be established according to circumstantial evidence, and implementing an administrative rather than adversarial judicial process, would not require any amendment to the law’s provisions. The law does not guide the Committee of Experts regarding these issues, and therefore the Committee need only change the method of discussing claims, both substantively and procedurally.

Amendments to the legislation which could promote its objectives would include repealing the choice of jurisdiction provision, requiring publication of cases that ended in favor of compensation (settlements included), increasing compensation sums and prolonging the deadline to file lawsuits. Regarding the choice of jurisdiction provision, it should be noted that there are alternatives in similar laws, which can be adopted in Israeli law as well. For example, according to U.S. law [26], petitioners must first file claims under the Vaccine Injury Compensation Program, but if the claim is dismissed or if the petitioner finds the compensation sum insufficient, she may file a civil tort claim [34, 35]. British law is even more lenient and allows vaccine injury claims to be brought in court at any time, albeit providing that any compensation already paid under the compensation law would be deducted from future awards [13]. The Israeli legislature intended to assist vaccine recipients who suffered injuries, but was concerned about the procedural difficulty of taking into account previously awarded sums in any additional legal proceedings. It seems that this challenge could be solved by granting the option of suing for damages under torts law after the claimant has already exhausted his or her rights under the Vaccine Injury Compensation law. The torts claim option may prevent the feeling of being “silenced”, which informants in this study described, and promote a sense of fairness, solidarity and trust.

As for the publication of cases in which compensation was awarded and concerns that knowledge of this compensation might deter the public from getting vaccinated, full transparency regarding compensation payments to vaccine recipients who suffered injuries attributed to the vaccine, accompanied by clarifications of the related scientific issues, should lead to deeper trust rather than deterrence.

In conclusion, the study’s findings show that the objectives of the Vaccine Injury Compensation Law have not been achieved. The law’s implementation fails to compensate vaccine recipients who suffered injuries attributed to the vaccine in an expeditious and efficient procedure, and as a result, the public health principles underlying the law, including fairness and solidarity, remain purely theoretical. In addition, the adversarial proceedings as well as some of the law’s provisions have the potential to undermine public trust in the State’s willingness to grant compensation for injuries that are attributed to vaccines and thereby subvert the law’s pivotal objective of promoting trust and vaccine compliance.

The apparent liability exemption, which excludes the possibility of redress from the Covid-19 vaccine manufacturers in case of injuries that are attributed to the vaccine should be accompanied by law amendments and changes in the method of deliberating claims so as to ensure the possibility of appropriate and timely redress in the event of injury.

The required law amendments are repealing the choice of jurisdiction provision, requiring publication of cases that ended in favor of compensation, increasing compensation sums and prolonging the deadline to file lawsuits.

However, the most important required change is in the method of discussing claims, both substantively and procedurally. Deciding that causal connection can be established according to circumstantial evidence, and implementing an administrative rather than adversarial judicial process, would not depend upon any amendment to the law’s provisions and can be implemented by the appointed expert committee.

In light of the fact that the vaccination issue in its entirety (eligibility for vaccines, funding, supply, compliance promotion strategies) is not regulated by Israeli legislation, amendments to the Vaccine Injury Compensation Law and implementation of the law in a manner that is consistent with its objectives should be incorporated into comprehensive legislation on vaccine policy.

## Data Availability

The datasets used and analyzed during the current study are available from the corresponding author on reasonable request.

## Appendix

General guideline topics:

- Presentation of the research topic and its objectives, presentation of the interviewer, ensuring informant’s anonymity, consent request to record and transcribe the interview, clarification that the purpose of the interview is to obtain information and gain insight into the topic.
- Informant’s professional background and acquaintance with the Vaccine Injury Compensation Law—a descriptive question aimed at elucidating the informant’s point of view.
- What is your view on the fact that a small number of vaccine recipients who had suffered injuries received compensation under the law? Why do you think this is so? Responsive questions aimed at a critical examination of the law.
- In your view, what criteria should guide the Committee of Experts in determining the existence of a causal link? Emphasis: legal causality versus epidemiological causality; circumstantial evidence versus an accurate description of the mechanism of injury. Evaluation/opinion question aimed at discussing the issue of causality and its impact on the law’s application.
- What is your view on the compensation sums stipulated in the law? Evaluation/opinion question whose goal is to clarify opinions on the effect of remediation stipulated in the law on the law’s application.
- How do you think claims for vaccine injury compensation should be deliberated? Evaluation/opinion question. Emphasis: Adversarial method versus administrative method.
- What ethical dilemmas do you think arise during committee deliberations? Evaluation/opinion question aimed at critical examination of the law’s application.
- What challenges are faced by vaccine of vaccine recipients who had suffered injuries seeking compensation for injuries? Open question.
- What changes do you think should be made to the law? Evaluation/opinion question

## Key points

- The Vaccine Injury Compensation Law, 1989, did not achieve its goals of aiding vaccine recipients who had suffered injuries in obtaining compensation in an expeditious and efficient procedure and promoting vaccine compliance.
- The requirement for scientific evidence for causality, the adversarial deliberations, and some of the law’s provisions, might undermine public trust in the State’s willingness to compensate vaccine recipients who had suffered injuries.
- Deliberating claims in an administrative procedure, relying on circumstantial evidence as to an association between vaccine and an injury, and amendments to the law’s provisions, may aid injured individuals, and improve public trust in health authorities in general and in vaccine policy makers in particular.
